# Comprehensive Characterization of 10,571 Mouse Large Intergenic Noncoding RNAs from Whole Transcriptome Sequencing

**DOI:** 10.1371/journal.pone.0070835

**Published:** 2013-08-12

**Authors:** Haitao Luo, Silong Sun, Ping Li, Dechao Bu, Haiming Cao, Yi Zhao

**Affiliations:** 1 Bioinformatics Research Group, Advanced Computing Research Laboratory, Institute of Computing Technology, Chinese Academy of Sciences, Beijing, China; 2 National Heart, Lung and Blood Institute, National Institutes of Health, Bethesda, Maryland, United States of America; University of California, Davis, United States of America

## Abstract

Large intergenic noncoding RNAs (lincRNAs) have been recognized in recent years to constitute a significant portion of the mammalian transcriptome, yet their biological functions remain largely elusive. This is partly due to an incomplete annotation of tissue-specific lincRNAs in essential model organisms, particularly in mice, which has hindered the genetic annotation and functional characterization of these novel transcripts. In this report, we performed *ab initio* assembly of 1.9 billion tissue-specific RNA-sequencing reads across six tissue types, and identified 3,965 novel expressed lincRNAs in mice. Combining these with 6,606 documented lincRNAs, we established a comprehensive catalog of 10,571 transcribed lincRNAs. We then systemically analyzed all mouse lincRNAs to reveal that some of them are evolutionally conserved and that they exhibit striking tissue-specific expression patterns. We also discovered that mouse lincRNAs carry unique genomic signatures, and that their expression level is correlated with that of neighboring protein-coding transcripts. Finally, we predicted that a large portion of tissue-specific lincRNAs are functionally associated with essential biological processes including the cell cycle and cell development, and that they could play a key role in regulating tissue development and functionality. Our analyses provide a framework for continued discovery and annotation of tissue-specific lincRNAs in model organisms, and our transcribed mouse lincRNA catalog will serve as a roadmap for functional analyses of lincRNAs in genetic mouse models.

## Introduction

Noncoding RNAs (ncRNAs) are transcripts that do not encode proteins or peptides, yet which play a variety of structural or regulatory roles in biological processes. Several major classes of ncRNAs, including ribosomal RNAs, small nucleolus RNAs and microRNAs (miRNA), have been extensively characterized and their functions have been well established [1]. For example, miRNAs have been recognized as key regulators through which cells fine-tune their proteomes and they have been implicated in nearly every important signaling and metabolic pathways. Altered miRNA profiles are linked to a number of pathological conditions, while multiple miRNAs are currently being evaluated as potential therapeutic agents for disease [1], [2].

In recent years, significant advances in sequencing technology have expanded the RNA world even further [3]. A group of noncoding RNAs, large intergenic noncoding RNAs (lincRNAs), have emerged as a major uncharacterized territory of the mammalian transcriptome [4], [5]. These transcripts are larger than 200 bases and they are transcribed from intergenic regions. A few ubiquitous features of lincRNAs have been uncovered in efforts devoted to cataloguing and annotating lincRNAs in human genome, and a limited number of lincRNAs have been studied in depth in order to identify their functions [6], [7], [8], [9]. However, since these novel transcripts comprise over half of the transcriptional units (TUs) in mammalian genomes [10] and their expressions are often dynamically regulated, the current annotations of lincRNAs are far from complete, thus limiting the extent of bioinformatics analyses that can be performed, and hindering the establishment of a unified model of their regulation and mechanisms of action. For example, several computational methods have been developed to reconstruct lincRNA transcriptome [11], [12] yet most of them have only been applied to limited number of species and often only to humans. In light of the reported lower evolutionary conservation of lincRNAs, the efficiency of these methods must be validated in other species to create a universal approach for lincRNA assembly, which could significantly accelerate lincRNA discovery while at the same time allowing in-depth comparative analyses of noncoding transcripts between species. For example, though mice represent the most widely utilized model organism for genetic elucidation of genes implicated in human pathologies, a comprehensive catalog of tissue-specific lincRNAs in mice is still lacking, and an efficient lincRNA assembly pipeline has yet to be established.

In this study, we carried out *ab initio* assembly of mouse lincRNA transcriptome across multiple tissues and we identified 3,965 novel lincRNA genes that have no overlap with currently known coding and noncoding transcripts. In combination with all know lincRNAs, we established an inclusive catalog of mouse lincRNAs. We also systemically analyzed all lincRNAs in our collection to map their key global features and to analyze their evolutionary conservation. Finally, we used a ‘two-color’ co-expression network method to assign functionalities to lincRNA groups and to determine how their potential expression correlates with that of neighboring coding genes. Since nearly one third of disease-associated SNPs are located in noncoding regions, our work not only establishes a roadmap for genetic analysis of lincRNAs in mice but it also provides a unique tool for scientists who perform disease modeling in this important model organism.

## Results

### Transcriptome reconstruction of the mouse tissues

**Figure 1:**
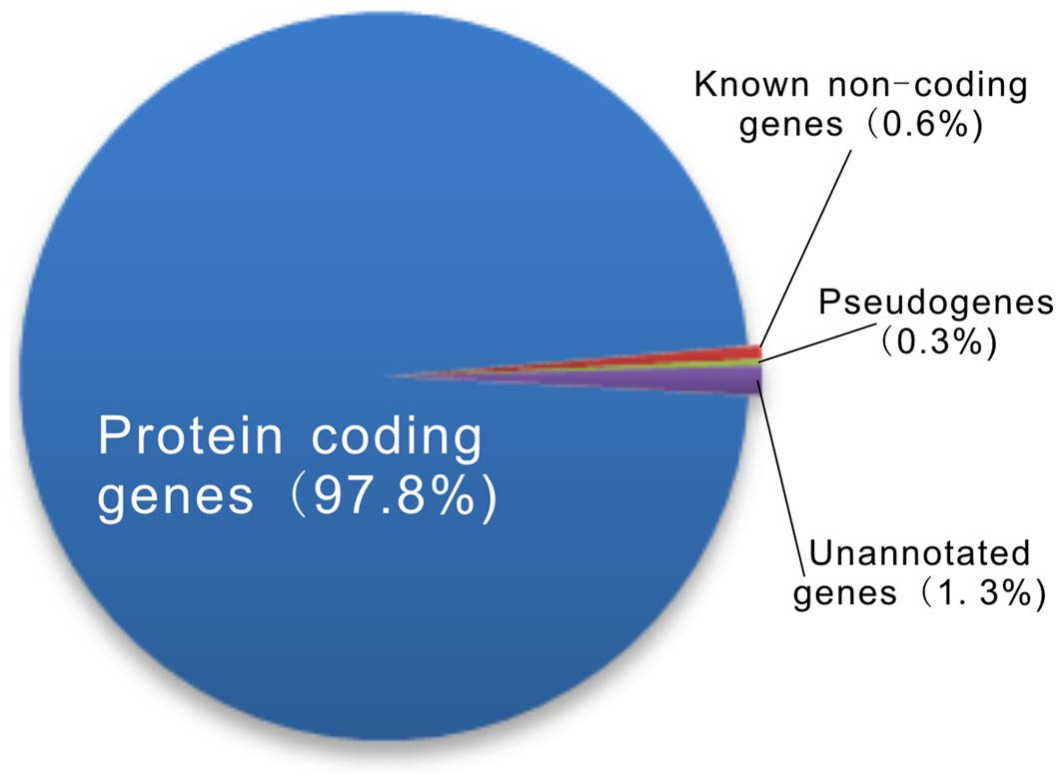
Statistics of mouse tissues transcriptome. The pie chart displays transcript distribution among protein coding, known non-coding, pseudogene, and unannotated genes.

The RNA-seq data used in this study were downloaded from the Wellcome Trust Sanger Institute. To prepare the sequencing data, RNAs were extracted from six biological replicates of six different mouse tissues including heart, hippocampus, liver, lung, spleen, and thymus and they were sequenced on an Illumina Solexa platform [13]. These reads were paired and both lengths were 76 nt. Starting from a total of 1.9 billion reads, we performed short-read gapped alignment using Tophat [14] and recovered 1.4 billion (75%) mapped reads (see more details in Table S1). We then used *ab initio* assemble software Cufflinks [12] and Scripture [11] to reconstruct the transcriptome for each tissue based on the read-mapping results. Transcripts reconstructed by these two assemblers were separately merged into combined sets of transcripts using the Cuffcompare utility provided by Cufflinks. After filtering for the exon number, transcript length and coverage, we obtained nearly 2,400,000 reliably expressed multi-exon transcripts longer than 200 nt for each sample. We compared these transcripts to major genomic database (Table S2) and classified the combined transcripts into several different subsets; the majority of the transcripts (97.8%) correspond to annotated protein-coding genes and a small portion of the transcripts are known noncoding genes (0.6%) and pseudogenes (0.3%). We also found that 1.3% of the transcripts had no overlap with annotated transcripts and were designated as unannotated (Figure 1).

To assess the robustness of these *ab initio* assemblers, we analyzed their performance on protein-coding and well-characterized noncoding genes. The annotated transcripts we reconstructed using Cufflinks cover 71% of RefSeq coding genes [15] fully or partially, and Scripture could assemble 68% of all RefSeq coding transcripts. In combination, the two assemblers fully or partially reconstructed 72% of Refseq coding genes, which similar to previous reported efficacies of these tools [16]. To evaluate the assemblers' performance on noncoding RNAs, we compared the ∼14,000 know noncoding transcripts to a comprehensive lincRNA database. Because none of currently available databases has a collection of all known noncoding RNAs, we built an inclusive database called NONCODE [17] by combining annotated mouse noncoding transcripts from RefSeq [15], UCSC [18] and Ensembl [19] as well as mouse lincRNAs reconstructed by Guttman et al. [11]. In all, there were 1,197 annotated ncRNAs in RefSeq, UCSC and Ensembl databases that could be fully or partially reconstructed, corresponding to 9,630 of our mouse tissue transcripts, and 1,577 transcripts in our datasets matched 251 mouse lincRNAs in Guttman's novel lincRNAs dataset. Furthermore, we evaluated the performance of these *ab initio* assemblers on Fantom noncoding genes. Considering that there is a high percentage (∼30%) of single exon transcripts in the Fantom v3 database [10], and that our combined sets of multi-exon transcripts have been filtered by exon numbers and transcript length, we only used the original unfiltered transcripts reconstructed by Cufflinks and Scripture to perform the assessment. Comparing Fantom noncoding genes with our unfiltered transcripts revealed that 10,674 of Fantom transcripts could be fully or partially reconstructed. These results strongly support that these assembly approaches could robustly and reliably reconstruct both coding and noncoding transcriptomes at a global level.

### Identification of novel mouse lincRNAs

**Figure 2:**
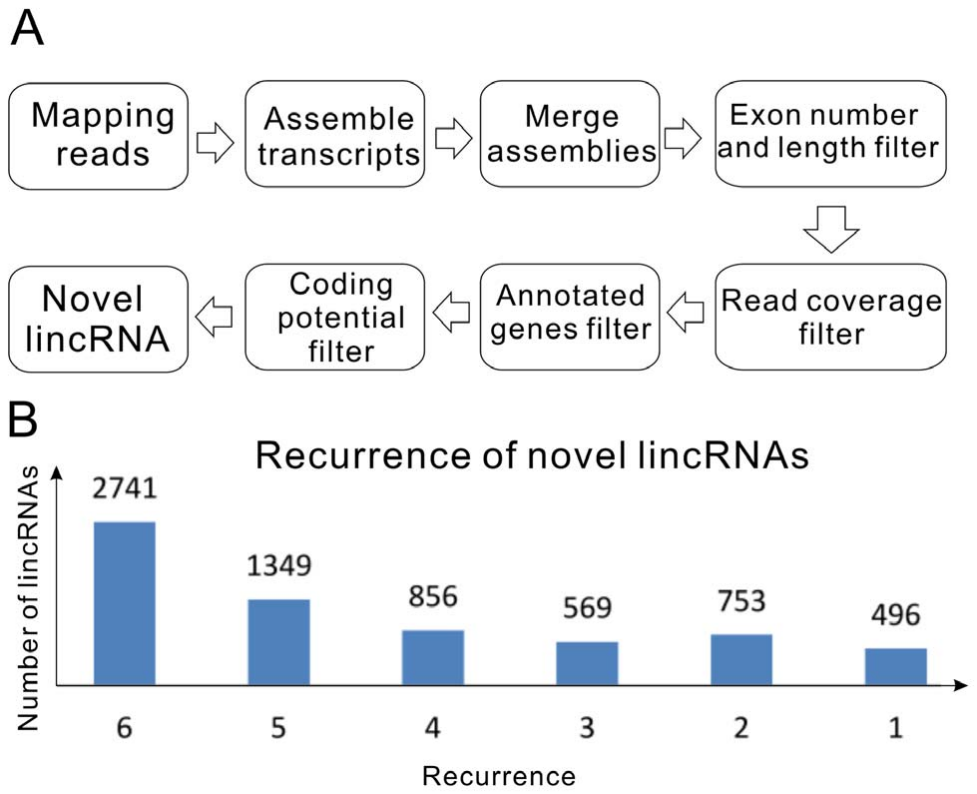
Identification of novel mouse lincRNAs. (A) Schematic overview of the novel lincRNA identification pipeline employed in this study. (B) Recurrence of lincRNAs in mouse tissues.

Based on the robust transcript reconstruction and broad availability of deep sequencing datasets, we have developed a novel lincRNAs detection pipeline system to identify novel lincRNAs that exhibit tissue-specific expression in mice (Materials and Methods, Figure 2A). We first analyzed the coding potential of unannotated transcripts using CPC [20] and CNCI in-house software filtering out 30% of all transcripts. Next, we focused only on intergenic transcripts that yielded 3,965 novel mouse lincRNA loci (6,764 transcripts) (Table S3). These transcripts had an average mature spliced size of 1.5 kb. Each transcript on average contained 2.5 exons of 620 nt long. In the novel lincRNAs dataset, about 48% were reconstructed by Cufflinks, 31% by Scripture, and 21% by both. These ratios are clearly lower than those of protein-coding genes for both programs, with which about 61% of genes can be reconstructed. This discrepancy might be caused by the different algorithms implemented by each assembler to reconstruct low-abundance transcripts, and similar observations have been reported in previous attempts to assemble low-expression transcripts using these programs [16], [21].

Since there were six biological replicates for each mouse tissue, we checked the recurrence of each individual novel lincRNA in our reconstruction to enhance our analyses. If a lincRNA transcript could be fully or partially reconstructed by Cufflinks or Scripture in one biological replicate of any tissue, we counted this as a recurrence. This recurrence test showed that about 40% of the 6,764 mouse novel transcripts could be reconstructed in all six biological replicates from at least one tissue, 20% of them in five biological replicates and only ∼7% transcripts recurred just once (Figure 2B). These results demonstrated that data generated by the two *ab initio* assemblers are highly reproducible, thus reducing the number of replicates has little effective to obtain reliable results.

### Characterization of tissue specific lincRNAs

**Figure 3:**
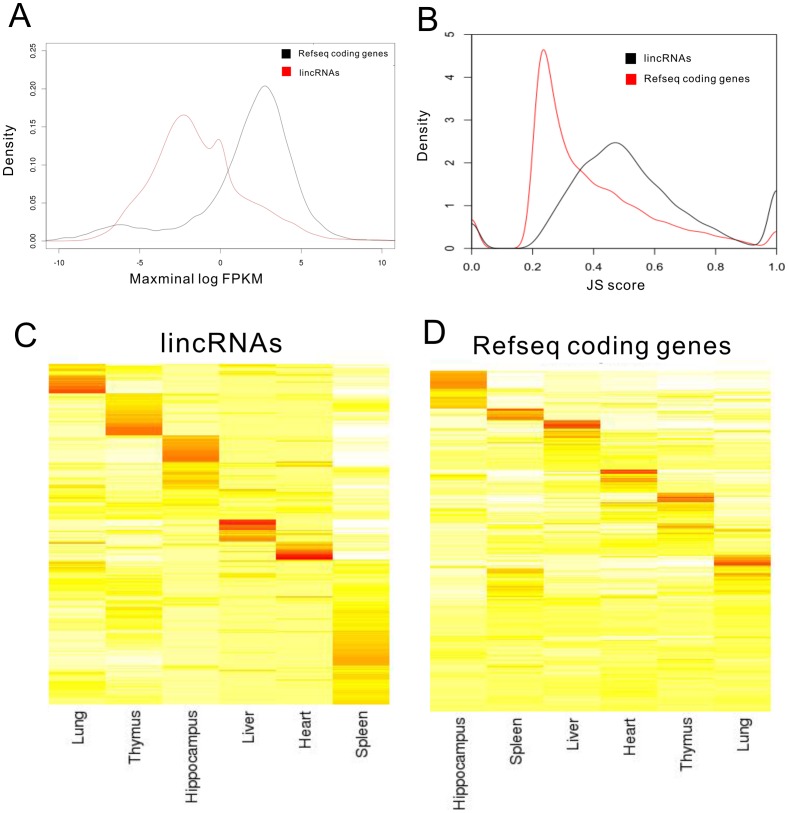
Tissue specificity of lincRNAs and protein-coding genes. (A) LincRNAs have lower expression levels than protein-coding genes. Maximal expression abundance (log2-normalized FPKM counts as estimated by Cufflinks) of each lincRNA (red) and coding (black) transcript across all tissues. (B) Tissue-specific expression. Shown are distributions of maximal tissue specificity scores calculated for each transcript across all tissues. (C) Cluster of the fractional density of lincRNAs across the row of log-normalized FPKM counts estimated by Cufflinks in each listed tissue. (D) Cluster of the fractional density of RefSeq protein-coding genes across the row of log-normalized FPKM counts estimated by Cufflinks in each listed tissue.

In combination with all know lincRNAs, we established a comprehensive catalog of 10,571 transcribed lincRNA genes (15,061 transcripts shown in Dataset S1). Based on the FPKM (Fragments Per Kilobase of transcript per Million mapped reads) of each transcript calculated by Cufflinks' “abundance estimation mode” across the six tissues, we compared the expression differences between lincRNAs and RefSeq protein-coding genes. The average expression level of lincRNAs was lower than protein-coding genes but lincRNAs also showed a wider range of abundance, with a subset of them equally abundant to mRNAs (Figure 3A). This pattern is consistent with previous studies [11], . We then calculated a tissue specificity score for each transcript using an entropy-based metric that relies on Jensen-Shannon (JS) divergence [16]. To assess the tissue specificity of mouse lincRNA expression, we calculated the Jensen-Shannon tissue specificity score (JS score) [16] for each transcript using the established procedure. Our analysis showed that distributions of JS scores for lincRNA and protein-coding genes are significantly different (*P* value of *Kolmogorov-Smirnov* test <10^−10^, Figure S1). Using JS score  =  0.5 as a cutoff, we demonstrated that the majority of lincRNAs (49%) are tissue-specific, relative to only 23% of protein-coding genes (Figure 3B-D and Datasets S2 and S3). Thus, mouse lincRNA expressions are clearly subject to tissue-dependent regulation either at the level of transcription or degradation.

**Figure 4:**
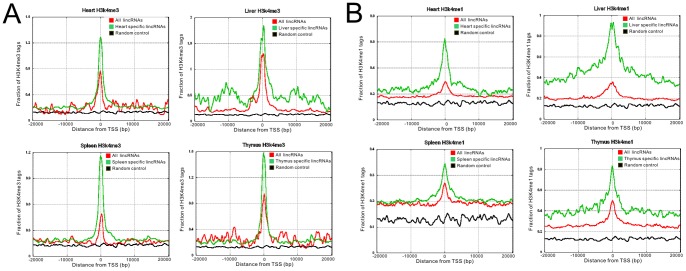
Histone marks of tissue specific lincRNAs (A) Trimethylation of lysine 4 of histoneH3 (H3K4me3) at promoter regions for heart, liver, spleen and thymus tissue. (B) Methylation of lysine 4 of histoneH3 (H3K4me1) at promoter regions. Red lines represent all lincRNAs, green lines represent tissue-specific lincRNAs and black lines represent random control.

Genes actively transcribed by RNA polymerase II often display trimethylation of lysine 4 (H3K4me3) or methylation of lysine 4 (H3K4me1) on histone H3 surrounding their promoter regions, and these active histone marks have been utilized to uncover lincRNAs from genomic regions that harbor no protein-coding genes. We investigated the chromatin states of lincRNAs in heart, liver, thymus and spleen to reveal that tissue-specific lincRNAs have highly enriched active histone marks surrounding their transcriptional start sites (TSS) compared to the rest of lincRNA pool (Figure 4). Therefore at least some of the tissue specificities of lincRNAs can be explained by enhanced transcription, and tissue-dependent histone modifications in the promoter may be used to predict the expression profiles of lincRNAs across different tissues.

### Conservation analyses of mouse lincRNAs

The evolutionary origin of a transcript often provides critical insight into its function. To assess the evolutionary conservation of lincRNA transcripts, we surveyed a catalog of mammalian and non-mammalian vertebrate transcripts that were syntenically mapped to the mouse genome. We found that 76% (11,479) of mouse lincRNAs have orthologous regions in the human genome (Dataset S4). Subsequently, using the TransMap tool to perform syntenic BLAST-Z alignments, we mapped all mouse lincRNAs to known transcripts across the vertebrate lineage. This analysis identified 1,477 lincRNAs syntenically paired with an orthologous transcript from TransMap (Dataset S4, Figure S2), accounting for 10% of all mouse lincRNAs. Trans-mapped lincRNAs also exhibit stronger tissue specificity and lower expression level relative to other lincRNAs. This moderate homology suggests that lincRNAs might be less conserved than their protein-coding counterparts although a quantitative assessment will require thorough analyses of datasets with higher sequencing depth across multiple species.

### Functional predication and neighborhood correlation of mouse lincRNAs based on the co-expression network

The comprehensive lincRNA catalog we constructed allows us to perform in-depth bioinformatics characterization of these novel transcripts. Here, we built a ‘two-color’ co-expression network to infer the putative lincRNA functions, using a method based on one we previously reported [23], [24]. In brief, FPKMs of lincRNAs and protein-coding genes were calculated across six tissues by the Cufflinks quantification module at individual gene level. To determine functional characteristics of lincRNAs, all FPKMs were further analyzed by a co-expressed module sub-networks method (Markov cluster algorithm, MCL) [23]. MCL is an efficient and powerful algorithm which identifies modules based on the simulation of random walks in a network. With default parameters (inflation value  = 1.8), the MCL algorithm found 51 functional enrichment modules with six or more genes, 32 of which consisted of both coding and lincRNA genes. Since each of these modules was significantly enriched for at least one GO BP term or KEGG pathway, we were able to functionally annotate 878 mouse lincRNAs based on the enriched GO associated with their modules (Datasets S5, S6). Our results indicated that a large portion of tissue-specific lincRNAs are potentially associated with critical developmental and metabolic processes including the cell cycle and cell development, and that they might be essential in maintaining each tissue's identity and functionality.

Furthermore, recent studies suggest that some lincRNAs may act in *cis* and regulate gene expressions within their chromosomal neighborhood [6], [25], although *trans* actions of lincRNAs in embryonic stem cells have also been clearly documented [26]. Our comprehensive catalog of mouse lincRNAs presents a unique opportunity to further explore this possibility. One expectation of the *cis* hypothesis is that the expression of lincRNAs and their neighboring genes would be correlated across our samples. Consistent with previous studies [6], [27], [28], lincRNA with protein-coding gene neighbors exhibits stronger positive correlations than neighboring coding genes with coding gene neighbors (Figure S3). To further determine whether lincRNA and protein-coding gene neighbors are co-regulated in the same functional context as strictly coding gene neighbors, we focused on the 878 functional annotated lincRNAs and their coding neighbors as described above, calculating their expression correlation coefficients and comparing GO terms associated with each. These results showed that 44% (388/878) of neighboring lincRNA-coding gene pairs have a correlation coefficient of 0.8, and 42% (164/388) of these are involved in the same biological process significantly (*P* value<10^−10^) more than expected by chance (among 10,000 randomly chosen gene pairs only 21% gene pairs shared the same GO annotations) (Figure S4, Dataset S7). These results suggest that a portion of lincRNAs might act locally to regulate their neighboring genes in *cis*. Vigorous bioinformatics analyses of large datasets as well as experimental testing will be required before this mechanism can be generalized to the majority of lincRNAs.

## Discussion

In this report, we presented the first comprehensive annotation of mouse lincRNAs based on whole transcriptome sequencing of multiple tissues and we provided in-depth analyses of these novel transcripts that lay the groundwork for further characterization of their pathophysiolocal consequences. We first reconstructed tissue-specific mouse transcriptomes from deep sequencing data to reveal a significant number of novel lincRNAs. The effectiveness of this approach is supported by the successful assembly of known protein-coding genes and lincRNAs and by the confirmed recurrence of novel lincRNAs in the majority of the six biological replicates for each tissue. We then calculated a tissue specificity score based on the FPKM for each transcript and demonstrated that mouse lincRNAs are expressed in a much more tissue-specific manner than protein-coding genes. The tissue specificity of lincRNAs is also reflected in the histone marks surrounding their transcriptional start sites (TSS), suggesting that lincRNAs share similar transcriptional signatures with protein-coding genes. Furthermore, we analyzed the conservation of lincRNAs across vertebrate species and revealed that lincRNAs have been under weaker selective constraints than protein-coding genes across mammalian and vertebrate ancestral genomes, which is consistent with previous reports based on other lncRNA catalogs [4], [27], [29]. Finally, utilizing a module based algorithm, we were able to predict putative functions for at least 878 lincRNAs, and we presented evidence supporting the hypothesis that lincRNAs might act in *cis* to affect expression in their chromosomal neighborhood.

Our work significantly complements the recent ENCODE publications [30]. The ENCODE project, which is an international effort to identify all regions of transcription, transcription factor association, chromatin structure, and histone modification in the human genome, has recently published 30 papers including a few that extensively characterize lincRNAs [27], [31], [32], [33]. However, ENCODE papers focus mainly on human samples which carry high degrees of genetic diversity and which often have very limited “true” biological replicates. On the other hand, the sequencing data used in our study were produced from mice of identical breeding with little genetic variance, as documented by the similarity of the six biological replicates provided. In addition, the strain of the two founder mice used in this study has been widely used to model human metabolic diseases, particularly obesity, diabetes, and cardiovascular disorders. A complete annotation of lincRNAs in this strain allows a unique opportunity for comparative analyses between humans and mice and it also provides an informatics resource to further validate the relevance of genomic variance in disease pathogenesis which is sought by the ENCODE project.

Our work also provides a framework for identifying and characterizing lincRNAs in other model organisms (Figure S5). Detailed annotations of genomes and transcriptomes of model organisms have proved to be instrumental in advancing almost all research areas of biology and the elucidation of lincRNA expression in model organisms will likely generate exciting new insights into how they function. Our lincRNA discovery pipeline can be easily adapted to study other model organisms and could help to annotate lincRNAs in these essential research subjects.

Most importantly, our work establishes a roadmap for scientists to study the physiological function of lincRNAs and to eventually pinpoint their pathological role in human disease. A number of human SNPs associated with disease have been mapped to lincRNA loci [34], [35] yet their causal relations with these pathological conditions have not been established. For mutations in coding genes, generating and characterizing a genetic mouse model is often the first step in establishing causality, but no mouse models with targeted knockouts of disease-associated lincRNAs have been widely adopted for study partly due to the incomplete annotation of mouse lincRNAs. Our work could fill this critical gap and is, in fact, already in practice in our current collaboration that aims to dissect the function of lincRNAs in physiology and disease in experimental mice.

## Materials and Methods

### RNA-seq data set

All RNA-seq data of mouse tissues used in this study were obtained from the Mouse Genomes Project at the Wellcome Trust Sanger Institute and can be directly downloaded from their website (accession number: ERP000591). Polyadenylated RNA-seq data utilized in this study were generated from six biological replicates of six mouse tissues including heart, hippocampus, liver, lung, spleen and thymus (the mouse strain used is a cross of C57BL/6J and DBA/2J). Each tissue yielded 54 million reads per sample on average, and the reads were paired and both lengths were 76 bp.

### Publicly available annotations

In this study we downloaded protein-coding genes of RefSeq [15], Ensembl [19], UCSC [18], and Vega [36] from the UCSC genome browser and all known noncoding genes from NONCODE 3.0 database [17] (Table S2).

### RNA-seq reads mapping

We used the spliced read aligner TopHat version V1.31 to map all sequencing reads to the mouse genome (mm9) [14]. Two rounds of TopHat mapping were used to maximize the splice junction information derived from all tissues. In the first round, all reads were mapped with TopHat using the following parameters: min-anchor = 5, min-isoform-fraction = 0, and the rest set as default; in the second round of TopHat mapping, all splice junctions produced by the initial mapping were collected and fed into TopHat to re-map each sample with the following parameters: raw-juncs, no-novel-juncs, min-anchor = 5 and min-isoform-fration = 0. Biological replicates of mapped reads from the same tissue were merged into a single BAM file to facilitate the transcript assembly and quantification.

### Transcriptome assembly

Aligned reads from TopHat were assembled into transcriptome for each tissue separately by both Scripture [11] or Cufflinks [12]. Both assemblers use spliced read information to determine exon connectivity, but with different approaches. Cufflinks uses a probabilistic model to simultaneously assemble and quantify the expression level of a minimal set of isoforms and provides a maximum likelihood explanation of the expression data in a given locus. Scripture uses a statistical segmentation model to distinguish expressed loci from experimental noise and uses spliced reads to assemble expressed segments. It reports all statistically significantly expressed isoforms in a given locus. The two approaches might generate different results in terms of assembled transcripts and numbers of products.

Cufflinks version V1.0.3 was run with default parameters (and ‘min-frags-per-transfrag = 0’) and Scripture version 1.0 was run with default parameters besides the omission of paired-end information to avoid conflicts that could occur while running Cufflinks abundance estimation mode in later steps.

### Novel lincRNAs detection pipeline

As expected from a mouse tissue cohort, individual transcript assembly may have noise from multiple sources such as artifacts generated by the sequence alignment, unspliced intronic pre-mRNA or genomic DNA contamination. To enhance the reliability of constructing expressed lincRNAs from mouse tissues, we developed an analysis pipeline to minimize noise and maximize recovery of “true hits” by implementing the following five steps: (1) Recalculate FPKM (fragments per kilobase of exons per million fragments mapped) and read coverage of each transcript across the six tissues separating transcripts as reliably expressed instead of background noise on the basis of FPKM using a trained decision tree; (2) Compare the combined transcripts with mouse coding genes with well-established databases such as Refseq [15], UCSC [18], Ensembl [19], Vega [36] for coding genes, and NONCODE for noncoding genes [17] and an independent Pseudogenes database [37] to eliminate transcripts that have at least one exon overlapping with any of them; (3) Calculate the coding potential of each transcript using CPC (coding potential calculator) [20] and CNCI (Coding Noncoding Index) in-house software to recover the transcripts which can be categorized as noncoding (CNCI, is a powerful signature tool that profiles adjoining nucleotide triplets to effectively distinguish protein-coding and non-coding sequences independent of known annotations; CNCI software is available at http://www.bioinfo.org/software/cnci); (4) Select transcripts that have more than one exon and which are longer than 200 bases; (5) Select the remaining transcripts that are located in the intergenic regions, at least 1 kb from any known protein-coding gene.

### Tissue specificity score and histone modification data

To evaluate the tissue specificity of a transcript, we devised an entropy-based method to quantify the similarity between a transcript's expression pattern and another predefined pattern that represents an extreme case in which a transcript is expressed in only one tissue [38]. All histone modification data were from mouse ENCODE data and were downloaded from UCSC Browser (http://hgdownload.cse.ucsc.edu/goldenPath/mm9/encodeDCC/wgEncodeLicrHistone/).

### Conservation analyses of mouse lincRNAs

We used the liftOver (http://genome.ucsc.edu/cgi-bin/hgLiftOver) tool to identify the orthologous locations of mouse lincRNAs in the human genome and used TransMap tools, which implements syntenic BLAST-Z alignments, to map all mouse lincRNAs to known transcripts across vertebrate linage.

## Supporting Information

Figure S1 **The distribution of JS score between lincRNAs (black line) and protein-coding genes (red line).**
(TIF)

Figure S2 **Orthologous transcripts of mouse lincRNAs in mammals and other vertebrates.** An example of mouse novel lincRNA conserved with Transmap transcripts between mouse, Human and Rat.
(TIF)

Figure S3 **Comparison of expression patterns between lincRNA:protein coding gene pairs (red line), coding:coding gene pairs (blue line) and random coding gene pairs (yellow line).**
(TIF)

Figure S4 **The distribution of correlation coefficient between 878 lincRNAs and their neighboring genes.** The portion of lincRNAs in five intervals of correlation coefficient are represented as different colors (left pie). The portion of lincRNAs who have high correlation (>0.8) and are involved in the same biological processes with their neighboring genes are also depicted (right pie).
(TIF)

Figure S5 **Data analysis framework of this study.**
(TIF)

Table S1 **Sample information and RNA-Seq statistics.**
(XLS)

Table S2 **All the annotated coding, non-coding and pseudogenes resource.**
(XLS)

Table S3 **Total transcript counts during the processing pipeline.**
(XLS)

Dataset S1 **The catalog of 10,571 transcribed lincRNA genes.**
(BED)

Dataset S2 **The list of JS scores of lincRNA genes.**
(XLS)

Dataset S3 **The list of JS scores of protein-coding genes.**
(XLS)

Dataset S4 **The orthologous regions of mouse lincRNAs in the human genome.**
(XLS)

Dataset S5 **The function prediction results of mouse lincRNAs.**
(RAR)

Dataset S6 **The list of GO terms according to the number of lincRNAs.**
(XLS)

Dataset S7 **The lincRNA and protein-coding gene pairs that shared the same GO annotations.**
(XLS)
