# Supplementary figures and images for: Comprehensive Characterization of 10,571 Mouse Large Intergenic Noncoding RNAs from Whole Transcriptome Sequencing
Source: PLoS One. 2013 Aug 12;8(8):e70835. doi: 10.1371/journal.pone.0070835 (PMC3741367; doi:10.1371/journal.pone.0070835)

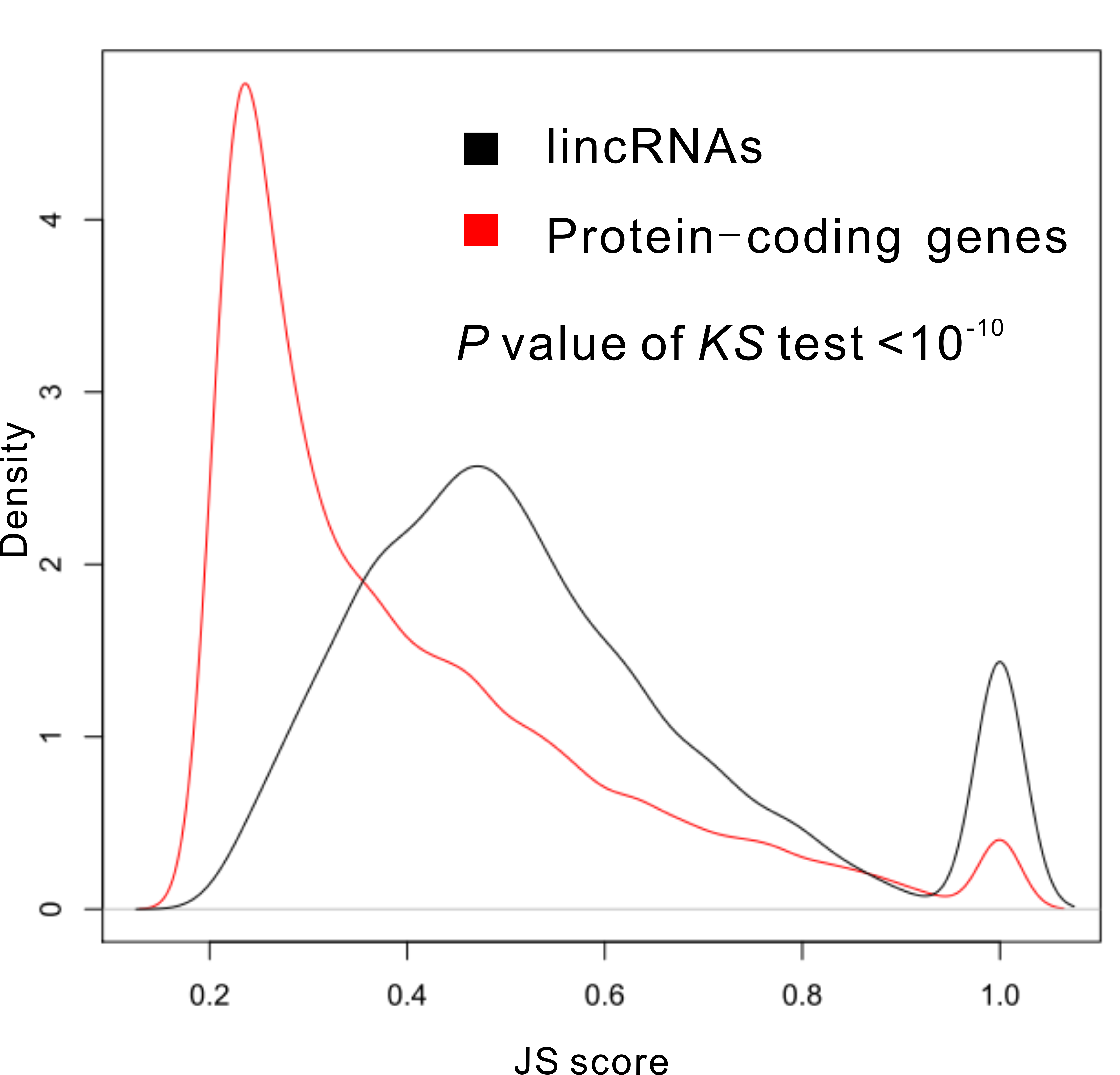

Supplement: Figure S1 — The distribution of JS score between lincRNAs (black line) and protein-coding genes (red line). (TIF) [file pone.0070835.s001.tif]

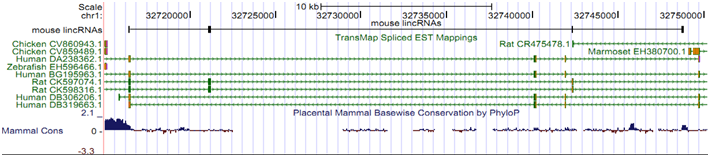

Supplement: Figure S2 — Orthologous transcripts of mouse lincRNAs in mammals and other vertebrates. An example of mouse novel lincRNA conserved with Transmap transcripts between mouse, Human and Rat. (TIF) [file pone.0070835.s002.tif]

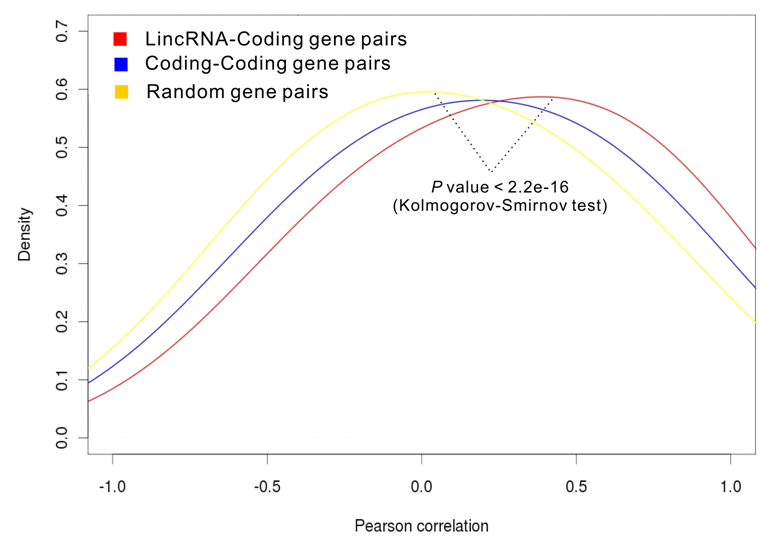

Supplement: Figure S3 — Comparison of expression patterns between lincRNA:protein coding gene pairs (red line), coding:coding gene pairs (blue line) and random coding gene pairs (yellow line). (TIF) [file pone.0070835.s003.tif]

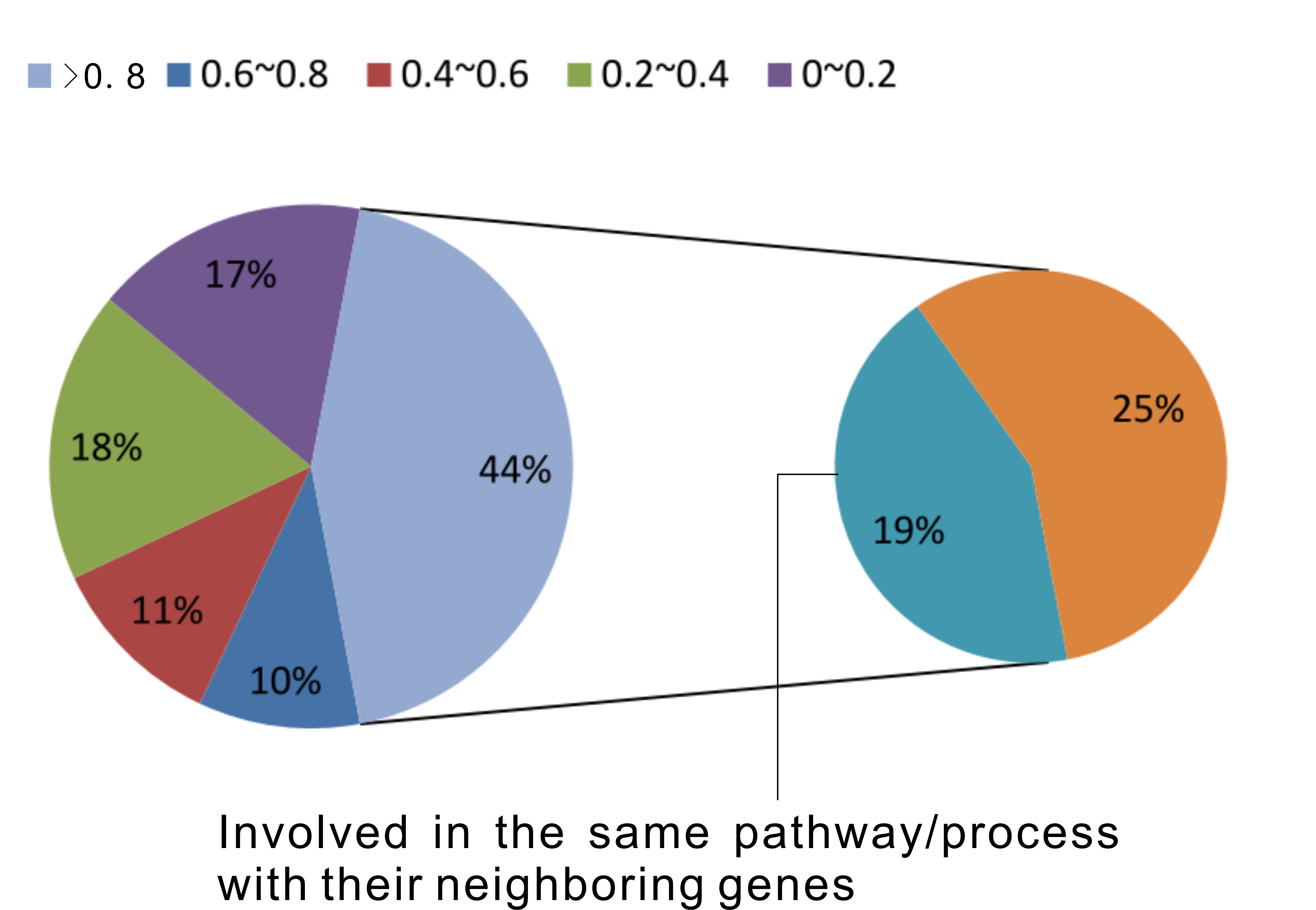

Supplement: Figure S4 — The distribution of correlation coefficient between 878 lincRNAs and their neighboring genes. The portion of lincRNAs in five intervals of correlation coefficient are represented as different colors (left pie). The portion of lincRNAs who have high correlation (>0.8) and are involved in the same biological processes with their neighboring genes are also depicted (right pie). (TIF) [file pone.0070835.s004.tif]

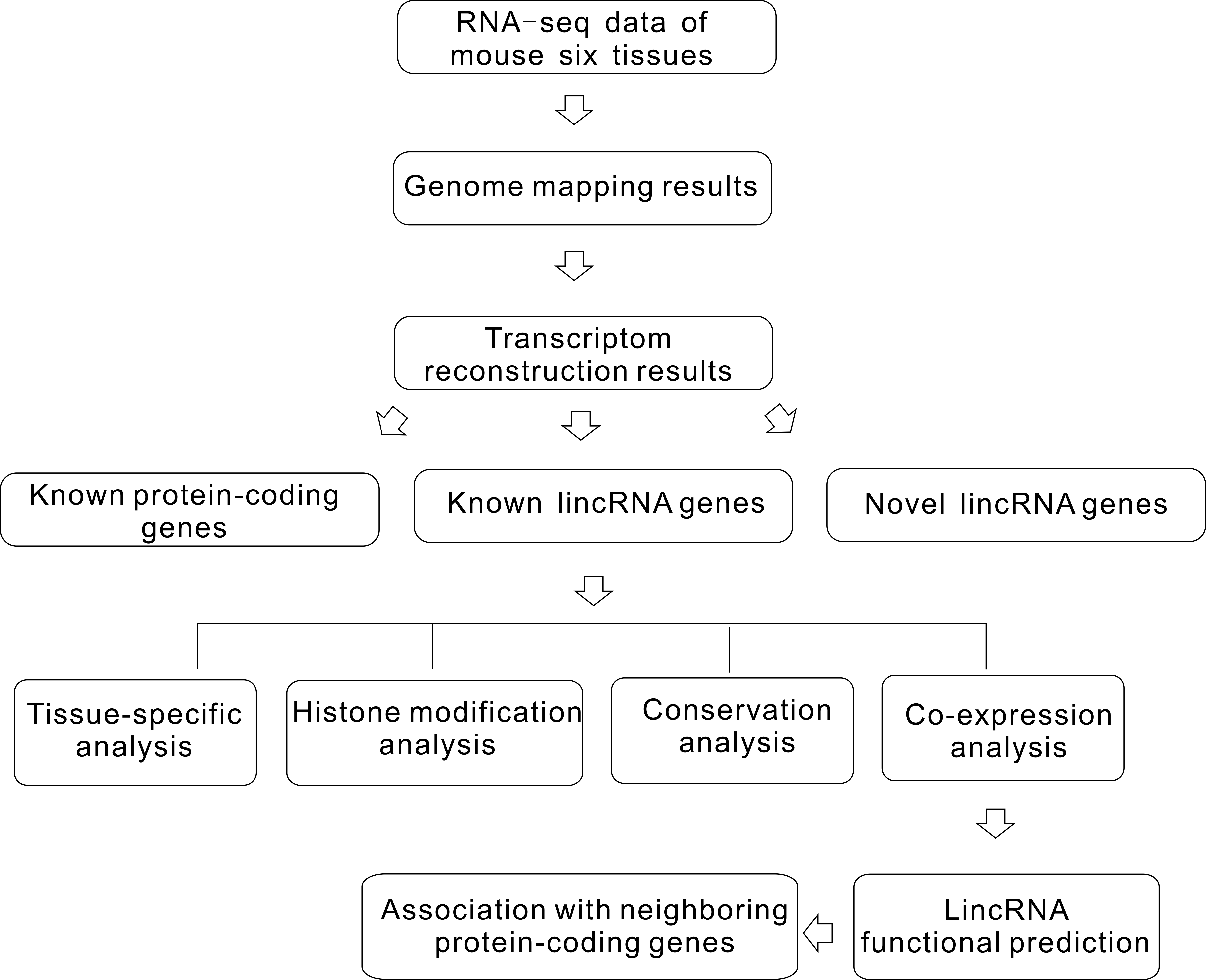

Supplement: Figure S5 — Data analysis framework of this study. (TIF) [file pone.0070835.s005.tif]
